# A 2:1 atrioventricular block in an adult patient with a Fontan circulation: from transesophageal pacing to echocardiographic guidance of epicardial pacemaker lead placement

**DOI:** 10.1016/j.ijcchd.2025.100580

**Published:** 2025-04-03

**Authors:** Jeff M. Smit, Madelien V. Regeer, Adrianus P. Wijnmaalen, Monique R.M. Jongbloed, Mark G. Hazekamp, Anastasia D. Egorova

**Affiliations:** a CAHAL, Center for Congenital Heart Disease Amsterdam Leiden, Leiden University Medical Center, Leiden, the Netherlands; b Department of Cardiology, Leiden University Medical Centre (LUMC), the Netherlands; c Department of Anatomy & Embryology, Leiden University Medical Centre (LUMC), the Netherlands; d Department of Cardiothoracic Surgery, Leiden University Medical Centre (LUMC), the Netherlands

**Keywords:** Atrioventricular conduction disorder, Congenital heart disease, Electrophysiological study, Epicardial pacemaker, Fontan circulation, Hypoplastic left heart syndrome

## Abstract

**Background:**

The diagnosis and management of atrioventricular (AV)-conduction disorders in patients with a Fontan circulation can be challenging. Little is known about the effects of various pacing strategies in single-ventricle patients. Here we report 1) the feasibility of transesophageal electrophysiological study (EPS) to assess AV-conduction in a patient with limited venous access and 2) the potential of echocardiography to guide epicardial systemic right ventricular (sRV) lead positioning and to evaluate the hemodynamic consequences of sRV pacing in order to mitigate long-term effects of single site ventricular pacing.

**Material and methods:**

A 21-year old male with hypoplastic left heart syndrome, palliated with Norwood and Glenn procedures, and ultimately extracardiac total cavopulmonary connection was seen for a regular check-up. He reported difficulty cycling against the wind. During exercise stress test, a 2:1 AV-block occurred at atrial frequencies >100 bpm with recovery of 1:1 AV-conduction at sinus rates of 80–100 bpm. In order to discriminate between a 2:1 conducted atrial tachycardia and an impaired anterograde AV-conduction during sinus rhythm in the setting of bilateral femoral vein and unilateral subclavian/jugular vein occlusion, EPS by transesophageal pacing was proposed.

**Results:**

Bipolar transesophageal pacing of the left atrium confirmed an anterograde AV-Wenckebach point at 103 bpm, confirming the indication for AV-sequential pacing. Epicardial leads were surgically placed on the atrium and sRV apex. During intraoperative sRV pacing, transesophageal echocardiography confirmed the ventricular contraction pattern to remain synchronous with stable estimated cardiac output. Transthoracic echocardiography was performed postoperatively to assess the effects of sRV pacing on ventricular (dys)synchrony, systolic function and estimated cardiac output. These parameters remained unchanged during sRV pacing, compared to intrinsic conduction, an important finding in light of preserving sRV function.

**Conclusions:**

EPS to assess AV conduction could safely be performed by transesophageal pacing in this patient with Fontan circulation. Moreover, echocardiographic guidance of epicardial sRV pacemaker lead placement was feasible and may help to define the optimal pacing site in Fontan patients.

## Abbreviations list

ACHD: adult congenital heart disease
AV: atrioventricular
CRT: cardiac resynchronization therapy
EF: ejection fraction
EP: electrophysiological study
FAC: fractional area change
GLS: global longitudinal strain
HLHS: hypoplastic left heart syndrome
NYHA: New York Heart Association
sRV: systemic right ventricle

## 1 Medical history

**Fig. 1:**
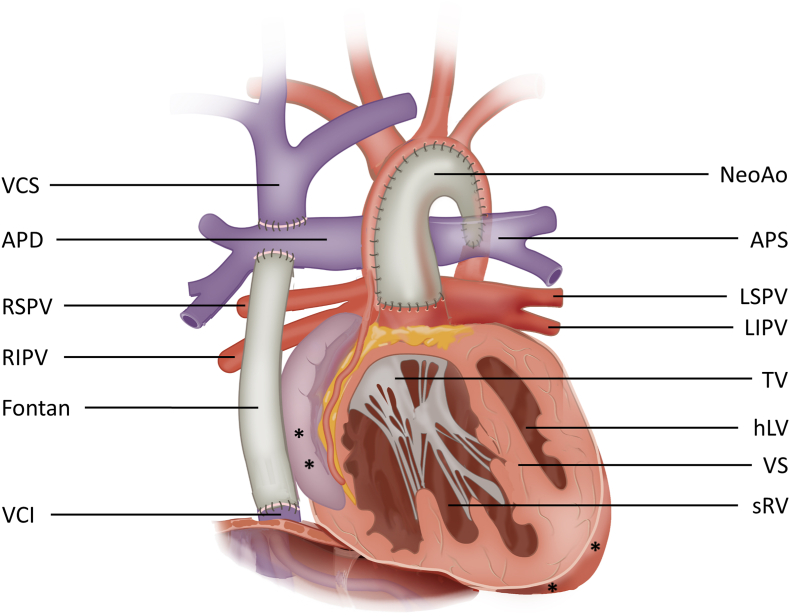
**Schematic overview of patient’s anatomy.** APD = arteria pumonalis dextra; APS = arteria pulmonalis sinistra; Fontan = extracardiac Fontan conduit; hLV = hypoplastic left ventricle; LIPV = left inferior pulmonary vein; LSPV = left superior pulmonary vein; NeoAo = neo-aorta with homograft patch to reconstruct aorta; RIPV = right inferior pulmonary vein; RSPV = right superior pulmonary vein; sRV = systemic right ventricle; TV = tricuspid valve; VCI = vena cava inferior; VCS = vena cava superior; VS = ventricular septum. ∗ indicates epicardial pacemaker leads placement.

A 21-year old male was seen in the outpatient clinic for adults with congenital heart disease (ACHD) for regular check-up. He was born with a hypoplastic left heart syndrome (HLHS) with mitral and aortic valve dysplasia and underwent a Norwood procedure as a first stage palliative approach at the age of 9 days. Two months later, a balloon angioplasty of a stenosis in the aortic arch was performed. At the age of 3 months, the aortopulmonary conduit (modified Blalock-Taussig shunt) was removed and a bidirectional cavopulmonary anastomosis (bidirectional Glenn shunt) was constructed. At the age of 2 years and 2 months, the Fontan circulation was completed with the creation of a non-fenestrated extracardiac total cavopulmonary connection (Goretex 16 mm) (Fig. 1). Concurrently, a valvuloplasty of the tricuspid valve was performed due to moderate-severe regurgitation. This was done by closing indentations in the septal leaflet and subsequently closure of the anteroseptal commissure. Two weeks later, a fenestration between the extracardiac Fontan conduit and right atrium was created due to severe systemic right ventricular (sRV) failure. Postoperatively, moderate residual tricuspid insufficiency was accepted. At the age of 3.5 years, the fenestration between the extracardiac conduit and right atrium was closed by a transcatheter intervention using an Amplatzer septal occluder. The clinical course was uneventful until early adulthood.

## 2 Clinical presentation and investigations

**Fig. 2:**
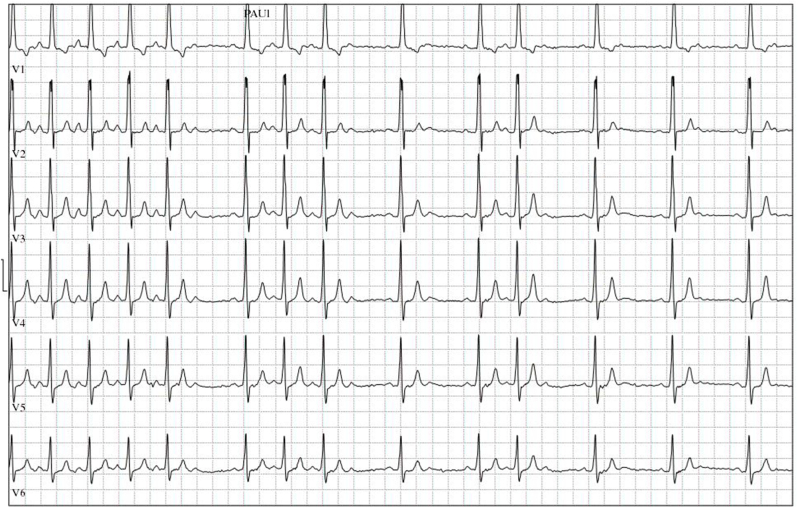
**Atrioventricular block during exercise stress test.** A 2:1 AV block occurred at atrial frequencies >100 bpm with recovery of the AV conduction at sinus rates of 80 to 100 bpm. Based on the 12-lead stress testing ECGs, discrimination between sinus tachycardia and 2:1 AV block during atrial tachycardia was challenging. AV = atrioventricular.

The patient reported difficulty cycling against the wind. Transthoracic echocardiography showed a mildly dilated and hypertrophic sRV with a moderate to good systolic function. sRV fractional area change (FAC), global longitudinal strain (GLS) and 3D ejection fraction (EF) were respectively 44 %, −14.6 % and 43 %. There was moderate tricuspid valve regurgitation and normal flow through the Glenn and Fontan conduits. During exercise stress test, there was a slight decline in maximal capacity compared to 1 year earlier (103 vs. 120 Watts, 42 % of predicted) and VO2 max (15.1 vs. 17.3 ml/min/kg, 35 % of predicted), with adequate peripheral oxygen saturation of 96 % at rest and 94 % at maximum effort. Notably, while previous maximal heart rate during exercise testing was 133–156 beats per minute (bpm), a 2:1 atrioventricular (AV) block now occurred at atrial frequencies >100 bpm with recovery of the AV conduction at sinus rates of 80–100 bpm (Fig. 2). Based on the 12-lead stress testing ECGs, discrimination between sinus tachycardia and AV block during an atrial tachycardia was challenging. The patient reported mild shortness of breath and palpitations at maximal effort during exercise testing and had had similar complaints when cycling to school in the previous months. Serum NT-proBNP levels were low (65 (<161) ng/L), kidney function was normal (creatinine 85 (<104) umol/L) and liver enzymes levels were elevated (gamma-glutamyl transferase 124 (<55) U/L, total bilirubin 29 (<17) umol/L and albumin 50 (<48) g/L). MRI study of the liver showed cardiac cirrhosis without signs of portal hypertension or hepatocellular carcinoma, which was consistent with the presence of Fontan-associated liver disease.

In patients with a Fontan circulation, augmentation of the cardiac output is driven primarily by the increase in heart rate [1]. Therefore, a proactive approach was pursued and it was decided to perform an electrophysiological study (EPS) to discriminate between an atrial tachycardia with a 2:1 conduction or an impaired anterograde AV conduction during sinus rhythm. The vascular access was limited by the occlusion of both the femoral, right subclavian and jugular veins. Diagnostic EPS was performed using transesophageal pacing (TEP) to determine antegrade AV conduction properties, not risking any damage to the only pertaining venous access to the left subclavian vein effluence. Bipolar left atrial pacing confirmed an anterograde AV Wenckebach cycle length of 580 ms (i.e. at 103 bpm, comparable with the heart rate frequency at which a 2:1 AV block was documented during the exercise stress test).

The patient was discussed in the ACHD heart team and subsequently accepted for dual chamber (DDD) pacemaker implantation with an epicardial atrial lead and one or two sRV leads, depending on intraoperative echocardiographic assessment of the systolic sRV function during pacing.

## 3 Management

**Fig. 3:**
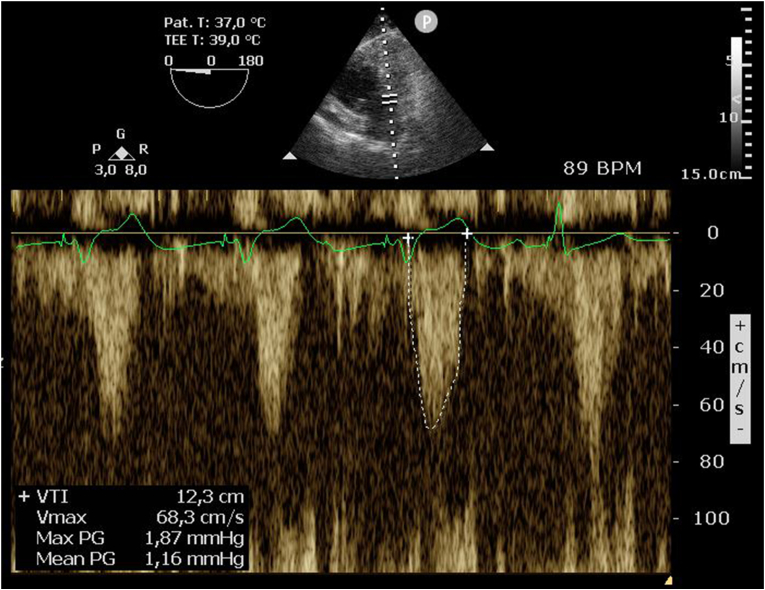
**Perioperative measurement of surrogates of cardiac output with and without sRV pacing using transesophageal echocardiography.** The RVOT VTI with and without sRV pacing was respectively 12.3 cm (at a heart rate of 89 bpm) and 14.7 cm (at 71 bpm). Max PG = peak pressure gradient; mean PG = mean pressure gradient; RVOT = right ventricular outflow tract; sRV = systemic right ventricle; Vmax = maximum velocity; VTI = velocity time integral.

**Fig. 4:**
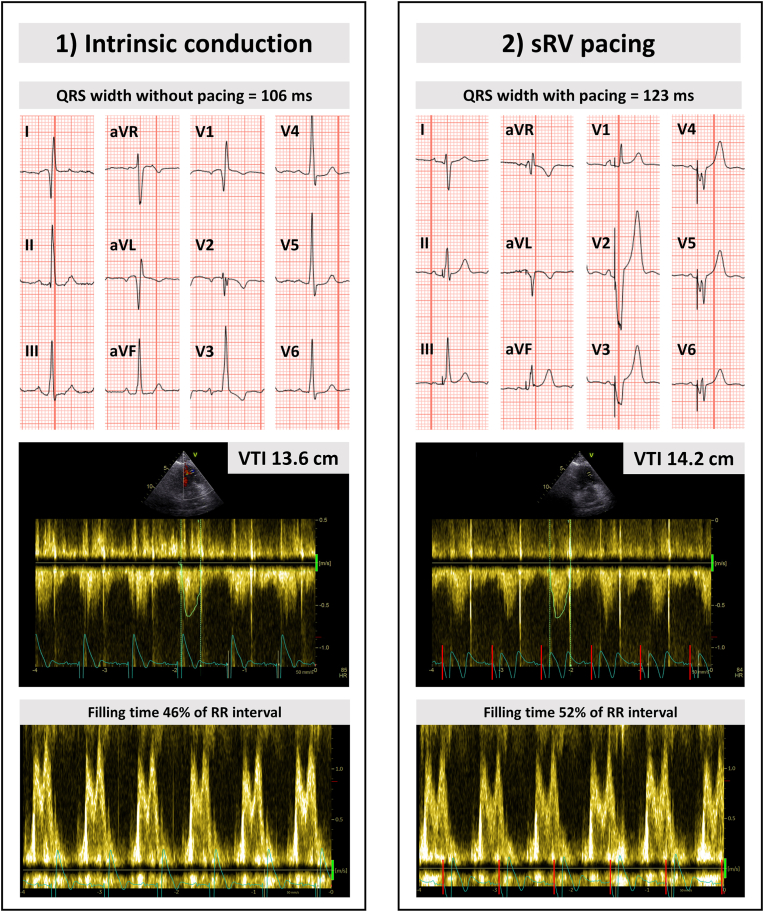
**Electrocardiographic and echocardiographic optimization during sRV pacing.** The effect of sRV pacing compared to intrinsic AV conduction on the sRV (dys-)synchrony, systolic function and estimated cardiac output was assessed using transthoracic echocardiography. During sRV pacing, the RVOT VTI was 14.2 cm and the E and A wave were partially fused with a diastolic filling time of 52% of the RR interval. During intrinsic AV conduction, the RVOT VTI was 13.6 cm and the E and A wave were partially fused with a diastolic filling time of 46% of the RR interval. The heart rate frequency remained stable at ∼85 bpm during the two different AV delay intervals. Compared to intrinsic conduction, sRV systolic function, synchrony and tricuspid insufficiency remained unchanged during sRV pacing. AV = atrioventricular; RVOT = right ventricular outflow tract; sRV = systemic right ventricle; VTI = velocity time integral.

The patient underwent a bilateral anterolateral thoracotomy without the use of cardiopulmonary bypass. Transesophageal echocardiographic evaluation of the sRV showed a synchronous ventricular contraction pattern with a moderate to good systolic function. Tricuspid valve regurgitation was only mild and probably underestimated due to the relatively low arterial blood pressure under general anaesthesia. First, a left-sided anterolateral thoracotomy was performed after which a bipolar epicardial lead (Medtronic Model 4968 CapSure Epi® Lead) was placed on the sRV apex. During sRV pacing, the ventricular contraction pattern remained synchronous and estimated cardiac output based on RVOT VTI remained relatively stable with and without pacing. The RVOT VTI with and without sRV pacing was 12.3 cm (at a heart rate of 89 bpm) and 14.7 cm (at 71 bpm), respectively (Fig. 3). Moreover, tricuspid valve regurgitation during sRV pacing was still only mild. The placement of 1 sRV lead at the apex proved to be sufficient and cardiac resynchronization therapy (CRT) was not indicated because of the relatively synchronous contraction due to apical pacing. Subsequently, a right anterolateral thoracotomy was performed and an epicardial lead was placed on the atrium. Due to extensive intrathoracic adhesions and thoracic venous collaterals due to the chronic occlusion of the right subclavian vein, tunnelling of the leads to the left thoracic wall was deemed to be at high risk of haemorrhagic complications. Therefore, the leads were tunnelled to the abdominal wall and connected to the pulse generator which was inserted under the left rectus muscle sheath. Three days after the procedure, transthoracic echocardiography was performed to assess the effect of sRV pacing compared to intrinsic AV conduction on the sRV (dys-)synchrony, systolic function and estimated cardiac output (Fig. 4). These parameters remained unchanged during sRV pacing, compared to intrinsic conduction, an important finding in light of preserving sRV function. Two days later, the patient was discharged from the hospital and had an uneventful recovery.

## 4 Follow-up

**Fig. 5:**
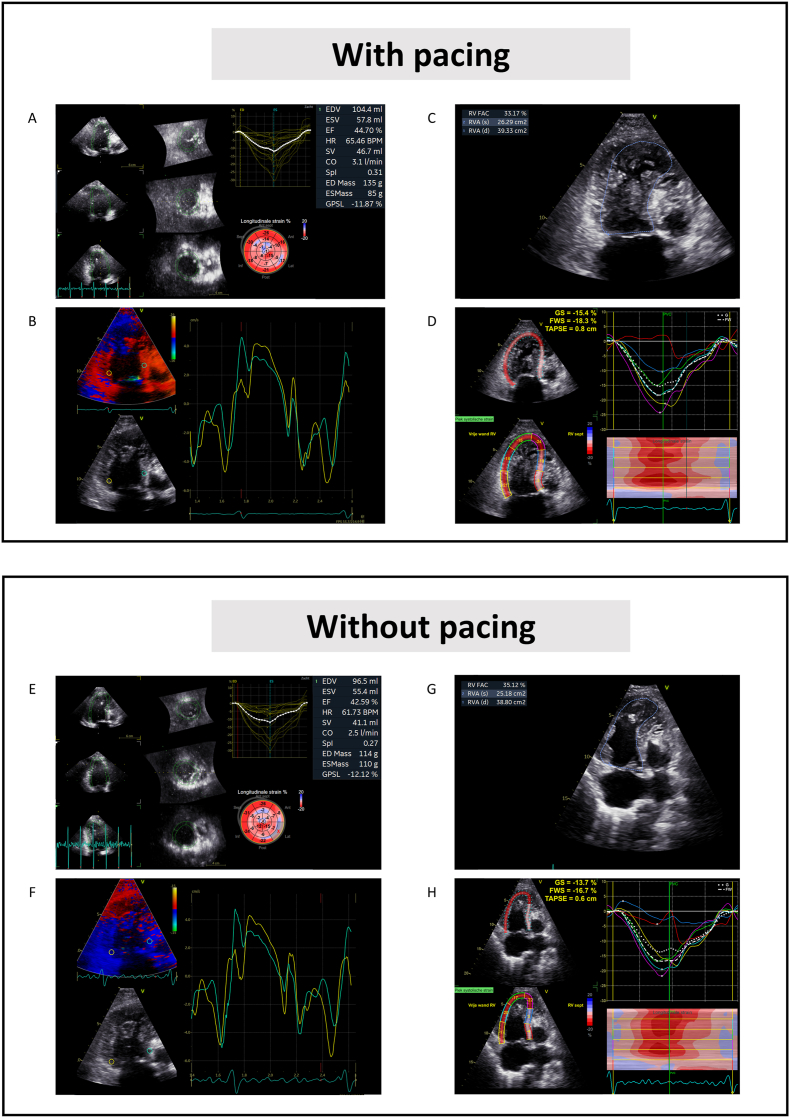
**Outpatient clinic echocardiography performed at 2.5 years of follow-up.** sRV systolic function remained unchanged compared to preoperative status (C and D with pacing and G and H without pacing). Although 3D regional volumetric curves showed only a slight delay with pacing compared to without pacing (A vs. E), no measurable septal-to-lateral delay was observed with tissue doppler imaging with and without pacing (B vs. F).

At 1.5 months of follow-up, the patient was still in New York Heart Association (NYHA) class II while there was a significant improvement in the patient's self-perceived exercise tolerance. Holter monitoring showed sinus rhythm with a maximum, minimum and mean heart rate of 145, 64 and 89 bpm, respectively. There was no occurrence of 2:1 AV block and ventricular paced rhythm was observed with sinus rate frequencies >105 bpm. Interrogation of the pacemaker revealed <1 % atrial pacing and 35 % sRV pacing. Outpatient clinic echocardiography at 2.5 years of follow-up showed a dilated and hypertrophic sRV with a mildly reduced function, moderate tricuspid regurgitation and an unobstructed flow through the Glenn and Fontan conduits. sRV systolic function remained unchanged compared to preoperative status, with an sRV FAC, GLS and 3D EF of 33 %, −15.4 % and 45 % with pacing and 35 %, −13.7 % and 43 % without pacing, respectively (Fig. 5). There was no progression of tricuspid regurgitation. Although 3D regional volumetric curves showed only a slight delay with pacing compared to without pacing, no measurable septal-to-lateral delay was observed with tissue doppler imaging with and without pacing.

## 5 Discussion

As a result of continuous developments in congenital heart disease surgery and catheter interventions, the prognosis of patients with single-ventricle congenital heart disease has radically improved over the past decades [2,3]. The introduction of the Fontan operation in 1968 has led to a significant improvement in survival, leading to a global growth in patients with a Fontan circulation to an estimated 70 000 patients in 2018 [4].

Sinus node dysfunction and high-grade AV block are two known complications after Fontan surgery requiring permanent pacemaker implantation. This can be the result from direct surgical trauma or developmental abnormalities of the sinus node or conduction system tissue [5,6]. In a Swedish national study including 599 patients who underwent a surgical Fontan procedure from 1982 to 2017, 78 patients (13 %) underwent a pacemaker implantation, most commonly due to sinus node dysfunction [7]. Of interest, although pacemaker implantation was less frequent in patients with HLHS (6 %), a relatively high proportion of these patients (50 %) received a pacemaker due to high-degree AV block.

The diagnosis and treatment of AV conduction disorders may be challenging, partially due to complex venous anatomy precluding adequate transvenous access in most Fontan patients. In patients with an extra-cardiac total cavopulmonary connection or ACHD patients with limited transvenous access due to multiple catheterizations or interventions, diagnostic EPS via TEP is a potentially useful method. By using this technique, antegrade AV conduction properties can still be reliably determined without risking any damage to the remaining venous access (if at all available). Moreover, patients with single-ventricle congenital heart disease requiring chronic ventricular pacing, are at increased risk of developing ventricular systolic dysfunction and secondary atrioventricular valve regurgitation [8]. In a study by Bulic et al., ventricular pacing in single-ventricle patients was associated with an increased risk of ventricular systolic dysfunction, atrioventricular valve regurgitation and heart transplantation or all-cause mortality at 7 years follow-up [9]. Nevertheless, if optimal ventricular lead placement was based on echocardiographic evaluation of ventricular (dys-)synchrony during intraoperative sRV pacing (as was performed in our patient), a more favourable outcome may be achieved.

In heart failure patients with NYHA class II or III, left ventricular systolic dysfunction and a wide QRS complex, cardiac resynchronization therapy (CRT) has been shown to reduce the risk of death and hospitalization for heart failure [10]. CRT, or dual-site ventricular pacing, could also be of benefit in single-ventricle patients requiring ventricular pacing. The effect of dual-site ventricular pacing in patients with Fontan physiology and high-grade AV block was studied by O'Leary et al. [11]. In total, 19 patients with dual-site and 43 patients with single-site ventricular pacemakers were reviewed with a median follow-up of 4.9 and 10.4 years, respectively. In the dual-site ventricular pacing group, the proportion of patients with ventricular systolic dysfunction did not change significantly over time (42 % vs. 37 %, P = 1.00). As opposed to our patient who's sRV systolic function remained stable during single-site ventricular pacing, ventricular systolic dysfunction was more frequently observed in the single-site ventricular pacemaker group during follow-up (10 vs. 33 %, P = 0.009). The fact that sRV systolic function did not deteriorate in our patient could possibly be explained by optimal lead placement being determined by the ventricular contraction pattern during intraoperative single-site pacing, in contrast to the patients included by O'Leary et al. It remains a matter of debate to what extend these findings can be extrapolated to the contemporary Fontan population and a prospective multicenter study with robust patient numbers would be required to determine the clinical benefit of dual-site ventricular pacing in univentricular heart patients.

In addition to determining on implantation of a single-vs. dual-site ventricular pacemaker, a decision has to be made on an atrial, ventricular or dual-chamber pacing strategy in Fontan patients with high-grade AV block. A study from Barber et al. suggests that atrial or dual-chamber pacing should be the preferred strategy over asynchronous ventricular pacing in Fontan patients [12]. Decision making for optimal pacing strategy is however based on individual characteristics, including the ventricular contraction pattern after ventricular single-site pacing, as was determined prior to implantation in the patient described in the current report. It should also be taken into account that obtaining adequate transvenous or epicardial access to both the atrium and systemic ventricle is likely to be very challenging, and implantation of an additional endocardial lead in single-ventricle patients with a Fontan circulation may pose an unproportional thromboembolic risk.

The current case demonstrates the potential of transesophageal echocardiography to guide epicardial sRV lead positioning. Echocardiographic parameters such as visual assessment of synchronicity of contraction pattern, systolic function, estimated stroke volume and cardiac output could be used to determine optimal sRV lead placement. Moreover, epicardial right atrial lead placement was feasible, enabling a dual-chamber pacing strategy leading to a better hemodynamic status by maintaining AV synchronicity.

## 6 Conclusions

EPS using TEP could be safely performed in a Fontan patient without adequate peripheral venous access to confirm the presence of an atrioventricular block. Moreover, echocardiographic guidance of epicardial sRV pacemaker lead placement is feasible and may help to define the optimal ventricular pacing strategy in Fontan patients. Despite chronic (>20 %) sRV apical pacing, ventricular (dys)synchrony, systolic function and estimated cardiac output remained unchanged during mid-term follow-up, an important finding in light of preserving sRV function.

## CRediT authorship contribution statement

**Jeff M. Smit:** Writing – original draft, Visualization, Project administration, Investigation, Formal analysis, Data curation, Conceptualization. **Madelien V. Regeer:** Writing – review & editing, Visualization, Supervision, Investigation, Conceptualization. **Adrianus P. Wijnmaalen:** Writing – review & editing, Investigation. **Monique R.M. Jongbloed:** Writing – review & editing, Visualization, Supervision, Resources, Conceptualization. **Mark G. Hazekamp:** Writing – review & editing, Investigation. **Anastasia D. Egorova:** Writing – review & editing, Visualization, Validation, Supervision, Resources, Investigation, Conceptualization.

## Funding

This research did not receive any specific grant from funding agencies in the public, commercial, or not-for-profit sectors.

## Declaration of competing interest

No conflict of Interest.
